# Removal of eDNA from fabrics using a novel laundry DNase revealed using high-resolution imaging

**DOI:** 10.1038/s41598-021-98939-0

**Published:** 2021-11-02

**Authors:** Hamish C. L. Yau, Adam K. Malekpour, Nazarmohammad G. Momin, Ana L. Morales-García, William G. T. Willats, Neil J. Lant, Catherine Y. Jones

**Affiliations:** 1 Procter and Gamble, Newcastle Innovation Centre, Whitley Road, Newcastle upon Tyne, NE12 9BZ UK; 2 School of Natural and Environmental Sciences, Newcastle University, Devonshire Building, Newcastle upon Tyne, NE1 7RU UK

**Keywords:** Biotechnology, Molecular biology

## Abstract

Washed textiles can remain malodorous and dingy due to the recalcitrance of soils. Recent work has found that ‘invisible’ soils such as microbial extracellular DNA (eDNA) play a key role in the adhesion of extracellular polymeric substances that form matrixes contributing to these undesirable characteristics. Here we report the application of an immunostaining method to illustrate the cleaning mechanism of a nuclease (DNase I) acting upon eDNA. Extending previous work that established a key role for eDNA in anchoring these soil matrixes, this work provides new insights into the presence and effective removal of eDNA deposited on fabrics using high-resolution in-situ imaging. Using a monoclonal antibody specific to Z-DNA, we showed that when fabrics are washed with DNase I, the incidence of microbial eDNA is reduced. As well as a quantitative reduction in microbial eDNA, the deep cleaning benefits of this enzyme are shown using confocal microscopy and imaging analysis of T-shirt fibers. To the best of our knowledge, this is the first time the use of a molecular probe has been leveraged for fabric and homecare-related R&D to visualize eDNA and evaluate its removal from textiles by a new-to-laundry DNase enzyme. The approaches described in the current work also have scope for re-application to identify further cleaning technology.

## Introduction

Throughout the stages of garment use, from wearing, laundering and drying and storage; deposition of human and environmental soils can lead to malodorous and dull looking garments^1–5^. This results from the predisposition of garments to accumulate such soil deposits over multiple wear cycles. Though initially invisible and individually insignificant, the soils eventually form a synergistic macromolecular complex composed of multiple extracellular polymeric substances (EPS)^6^. Most of these EPS components are produced by laundry-relevant micro-organisms, which go on to form a sessile community of cells referred to as biofilms^7,8^. Matrixes composed of EPS are reported to act as a scaffold to maintain the structural integrity and ensure the survival of these bacterial communities^9^. These EPS matrixes contain a highly adhesive mixture of extracellular DNA (eDNA), polysaccharides, glycoproteins, phospholipids, humic acids and other siderophores^10–13^. Furthermore, this EPS assembly acts as a bio-absorbing agent attracting other soils and polymers in the initial stages of bacterial adhesion. These adhesion properties have been studied on solid glass surfaces^14^, fish gills^15^ and on a hydrophobic surface that mimics polyester^6^.

Extracellular DNA is believed to significantly contribute to the adhesive properties of EPS matrixes during soil adhesion^6^. Although its presence is key for agglomeration with other EPS matrix components, eDNA only constitutes a small proportion of this aggregated mass^16^. Interestingly, studies have shown that the activity of an extracellular deoxyribonuclease (DNase I, E.C. 3.1.21.1) is an efficient strategy to disrupt and disperse microbial communities of marine origin^17^. Further, the addition of DNase I has also been shown to significantly affect structural integrity of EPS biopolymers within *Micrococcus luteus* biofilms^6^. New work has since described the introduction of DNase I to target eDNA on textiles, which was shown to bring qualitative appearance benefits for real consumer items^18^. Taken together, we now understand eDNA as a key component for adhesion of other EPS components and for supporting EPS matrix formation. Whilst it has previously been shown that targeting eDNA using DNase I is an effective tool for preventing malodor and improving visual appearance of laundry^18,19^; it would be invaluable to also have a method for visualizing the eDNA removal benefit of DNase technology on real items. Imaging approaches have previously utilized histological stains and lectins to label cells, proteins, lipids and polysaccharides in bio-aggregates^20–23^, but these reagents lack the specificity required to confidently image eDNA.

In this study, we utilized an immunofluorescence approach using a monoclonal antibody (mAb) highly specific to Z-DNA as a tool to visualize bacterial eDNA on textiles^24^. This methodology is an advancement on others that are based on DNA stains and overcomes current limitations of using such stains like methyl green (which is stabilized by cellulosic components of cotton)^25^ and PicoGreen (unable to cover autofluorescence signal from brighteners and endogenous DNA of certain cotton fabrics); which are not optimal for imaging studies on such laundry items. This study describes our use of an imaging capability that complements existing DNA quantification methods, to allow us to measure and also visualize distribution of remaining DNA across threads and yarns of fabric and discriminate between superficial “surface” and “deep” hygienic cleaning performance. The use of DNase enzymes to remove eDNA from textiles will improve the sustainability of garment washing by maintaining high performance of detergent formulations even at low temperature cleaning cycles^18,19^.

## Results

### The use of DNase I results in a quantitative reduction of eDNA remaining on textiles after washing

**Figure 1:**
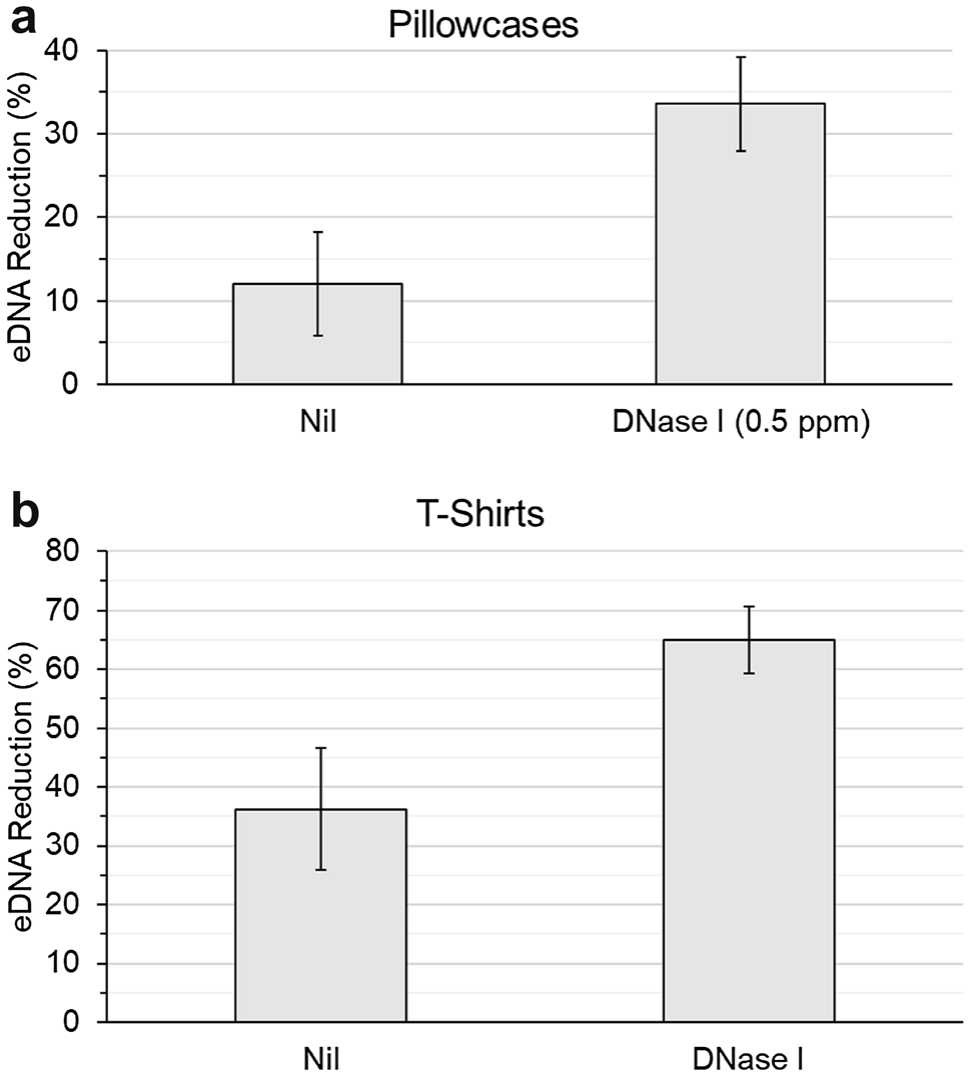
DNA quantification using PicoGreen shows there is a greater reduction in eDNA when washing soiled items in presence of DNase I. Percentage eDNA reduction on washed (**a**) pillowcases and (**b**) T-shirts, both show statistically higher removal of eDNA following wash with DNase I, compared to nil DNase I (S. Table 1—95% CI, n = 5).

Prior to any imaging work, we first carried out a quantitative assessment of eDNA removal from real consumer items following washing with DNase I. Split-item testing with consumer used pillowcases and T-shirts was carried out; whereby two halves of the same items were washed in Ariel 3 in 1 pods original (current base product) with or without the addition of 0.5 ppm active DNase I enzyme. Remaining eDNA from Nil enzyme vs DNase I washed items were extracted and quantified using PicoGreen (S. Table 1). Results showed that the percentage of eDNA reduction on pillowcases washed with DNase I (relative to an unwashed segment) was 34 ± 6%, compared to 12 ± 6% eDNA removal on Nil DNase I washed pillowcases (Fig. 1a). The extent of eDNA reduction was also statistically greater in T-shirts washed with DNase I. The relative percentage of eDNA reduction in T-shirts was 65 ± 6%, compared to only 36 ± 10% on the Nil DNase I washed half (Fig. 1b). These results suggest that addition of DNase I enzyme to a laundry wash does indeed reduce the relative percentage of eDNA remaining on real items.

### Molecular probe screening for detection of eDNA

**Figure 2:**
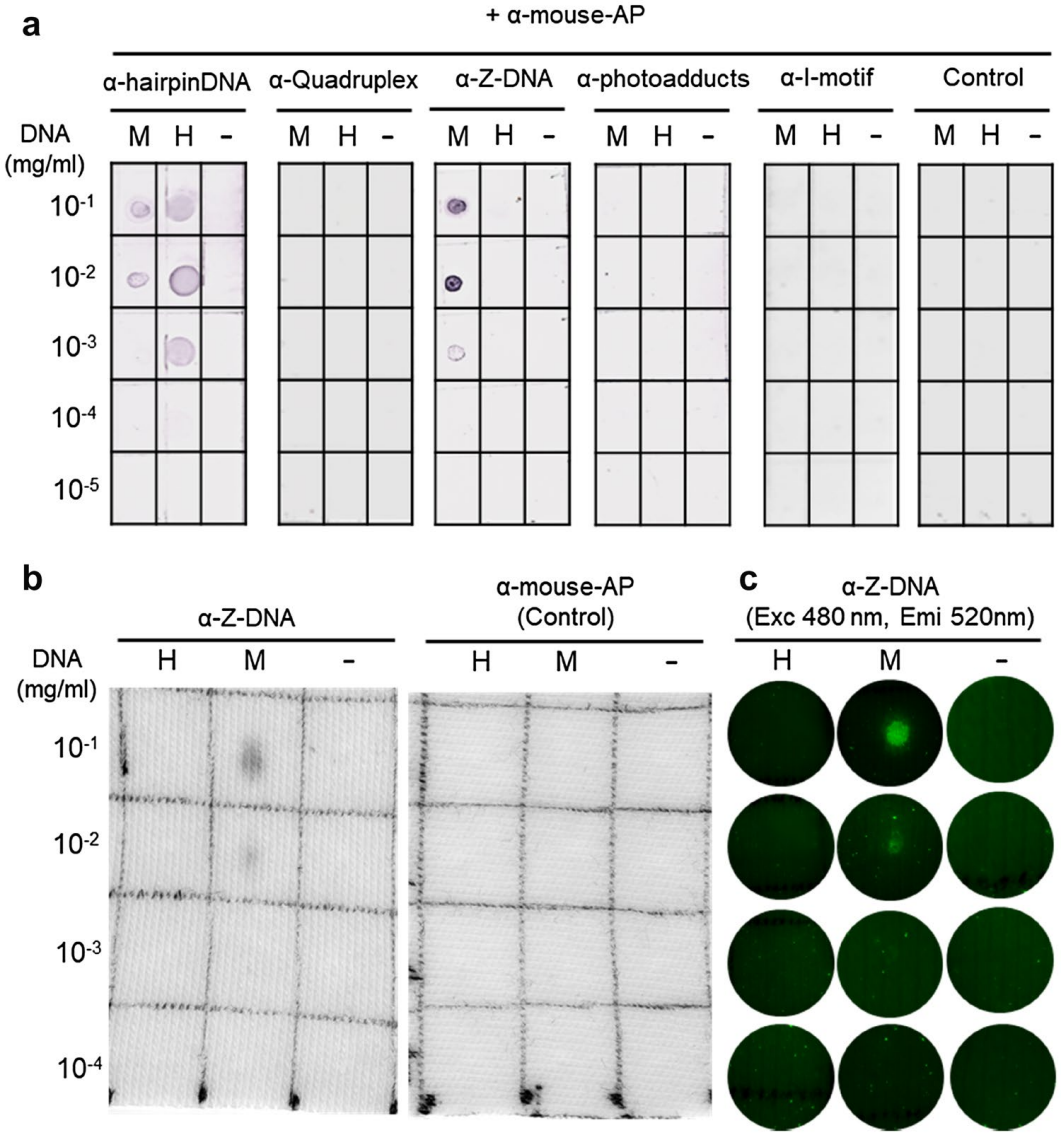
Z-DNA antibody binds microbial DNA. Testing antibody specificities against microbial and herring sperm DNA by immuno-dotblot assay, on (**a**) nitrocellulose membrane (AP, alkaline phosphatase detection). (**b**) Specificity of α-Z-DNA antibody tested against DNA spiked on cotton fabric (AP detection). (**c**) Binding of α-Z-DNA-Atto^488^ conjugate to DNA versions visualized using fluorescence microscopy. M, Microbial DNA (Sigma, D8259); H, Herring sperm DNA (Sigma, D6898); −, Control PBS.

To evaluate eDNA presence beyond general DNA content using PicoGreen (Fig. 1, S. Figure 1); we next looked to identify tools and optimize methods which would allow us to visualize removal of microbially-derived eDNA on textiles like cotton. With multiple antibodies available on the market but a lack of knowledge as to what structures of eDNA to expect on clothes, we selected five commercially available antibodies with specificities to a broad range of DNA epitopes. These included single stranded (ss) hairpin DNA, G-quadruplex DNA, Z-DNA, (6–4)-DNA photoadducts and Intercalated(I)-motif DNA. Utilizing an immuno(dot)blot assay, serial dilutions of microbial and herring sperm DNA (common model DNA that is easier to source than microbial DNA), were spiked onto a nitrocellulose membrane surface and the binding affinities of the respective antibodies evaluated. Antibodies recognizing DNA hairpins and Z-DNA were both able to bind microbial DNA down to concentrations of 0.1 mg/ml (Fig. 2a). Of the two candidates, α-Z-DNA specifically bound to microbial DNA but not herring sperm DNA (Fig. 2a). Importantly, the same binding pattern was observed when testing binding affinity of α-Z-DNA against microbial and herring sperm DNA spiked onto cotton fabric (Fig. 2b). Using an Atto^488^-conjugated version of α-Z-DNA, we were also able to observe the binding of the antibody to microbial DNA via fluorescence microscopy (Fig. 2c). The ability to detect binding of the Atto^488^-conjugated antibody to microbial DNA using epifluorescence microscopy demonstrated the feasibility of using this molecular probe to visualize removal of eDNA from fabric using DNase I.

### Imaging enzymatic removal of eDNA on real items

To assess whether the addition of DNase I reduced levels of microbially-derived-eDNA present on real items, we next imaged consumer-used terry towels and cotton T-shirts that had been washed in either Ariel 3 in 1 pods alone (Nil DNase I) or with DNase I added.

**Figure 3:**
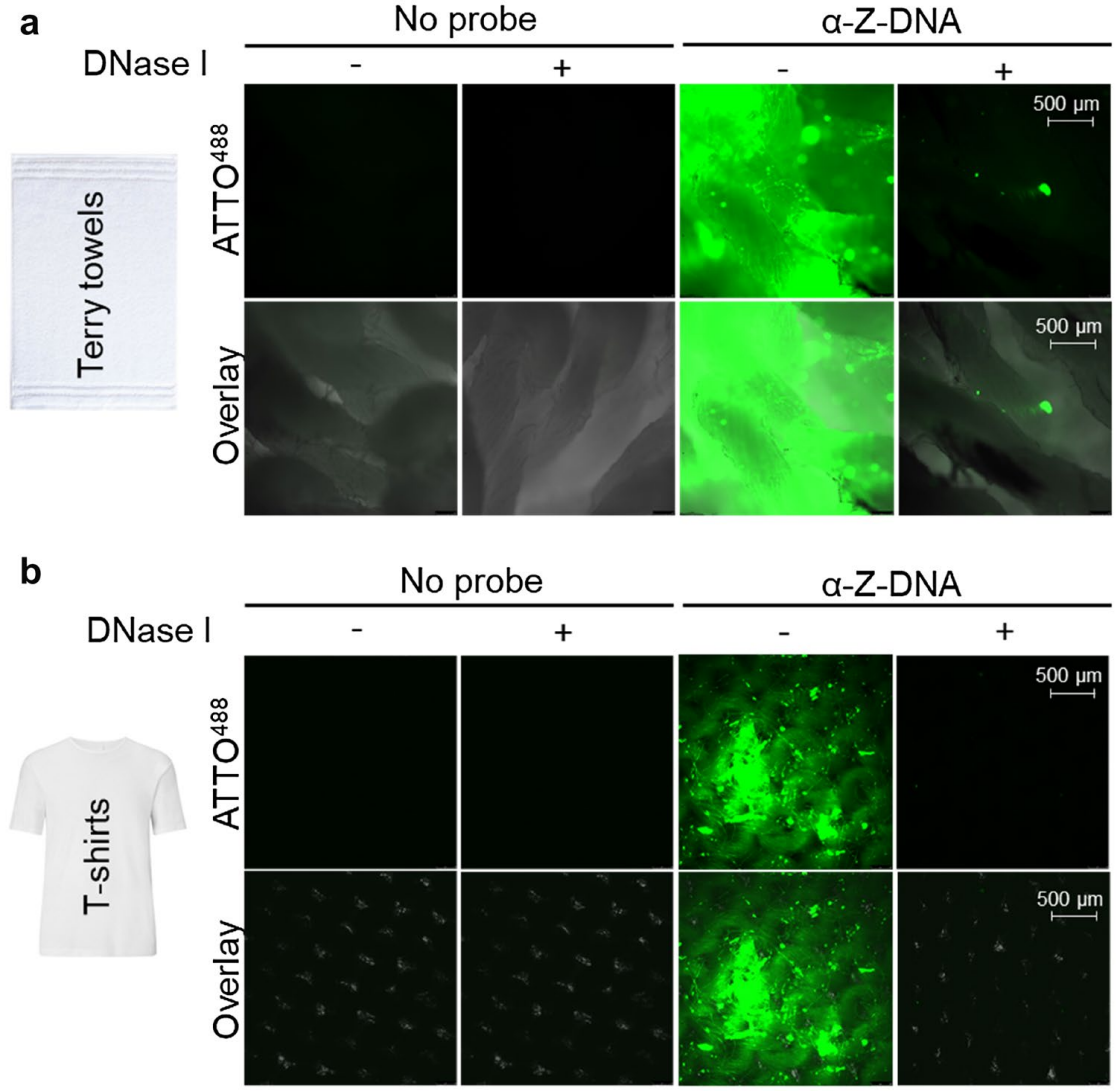
Washing real items in the presence of DNase I removes eDNA from soiled consumer items. Visualization of eDNA remaining on surface of soiled consumer (**a**) Terry towels and (**b**) T-shirts washed with and without DNase I, using α-Z-DNA-ATTO^488^ probe. Excitation/emission maxima 480/520 nm, respectively. Representative images of probed samples taken at ×5 magnification are shown. Further images shown in S. Figure 3. Controls correspond to autofluorescence of fabrics incubated without α-Z-DNA ATTO^488^.

The autofluorescence properties of the respective items as well as equivalent clean, unworn samples were analyzed with microscopy at 480 nm (excitation) and 520 nm (emission) wavelengths respectively. Under these conditions, using a 100–200 ms exposure time, both terry towel and T-shirt samples showed no discernible autofluorescence signal that would potentially interfere with our analysis of any subsequent probe binding (Fig. 3, S. Figure 2a).

When we analyzed towels and T-shirts probed with α-Z-DNA-ATTO^488^, there was visibly less fluorescence signal on the samples washed with DNase I, relative to the samples washed without (Nil) (Fig. 3, S. Figure 3). When quantified using Image J, there was an 74.5% decrease in fluorescence on towels washed with DNase I, relative to towels washed in Nil enzyme (S. Table 3). On T-shirts washed with DNase I, there was an 86.4% decrease in fluorescence signal remaining after washing, compared to T-shirts washed in Nil DNase I (S. Table 3); inferring that there was a lower level of microbial eDNA remaining for the probe to bind on the DNase I washed garment. This corroborates the quantitative reduction in eDNA remaining measured on DNase I washed samples (Fig. 1). Further because there was no fluorescence signal observed on a new unworn T-shirt after probing (S. Figure 2b), we reason that the eDNA signal that was observed was a result of consumer wearing and laundering cycles and is hence relevant to fabric-care.

To confirm specificity of probe binding to DNA; worn and washed T-shirt samples were incubated with commercially available DNA decontamination reagent (Decon) for 1 h, to remove all traces of DNA before being subjected to the same probing and imaging protocol as the rest of the probed T-shirts (S. Figure 2b). We found that a probed Decon treated swatch of a T-shirt, washed in Nil DNase I, displayed no fluorescence (S. Figure 2b). This was in stark contrast to the strong fluorescence observed on a paired, Nil DNase I washed, sample from the same T-shirt that had not been treated with the DNA removing chemical reagent (S. Figure 3b). Since the fluorescence signal was abolished following treatment with Decon, we reason that binding of the probe was indeed specific to deposited DNA present in the samples.

### DNase technology confers deep cleaning benefits

Having imaged the surface of cotton-containing garments, we next wanted to gain further insight into the ability of DNase I to remove DNA from deep fibers within cotton yarn, in particular within T-shirts. To do this, we prepared cross-sections of T-shirt samples washed with and without DNase I and imaged these using confocal laser scanning microscopy; to delve deeper into the fabric and image the surface and cross-sections of T-shirt samples washed with and without DNase I.

**Figure 4:**
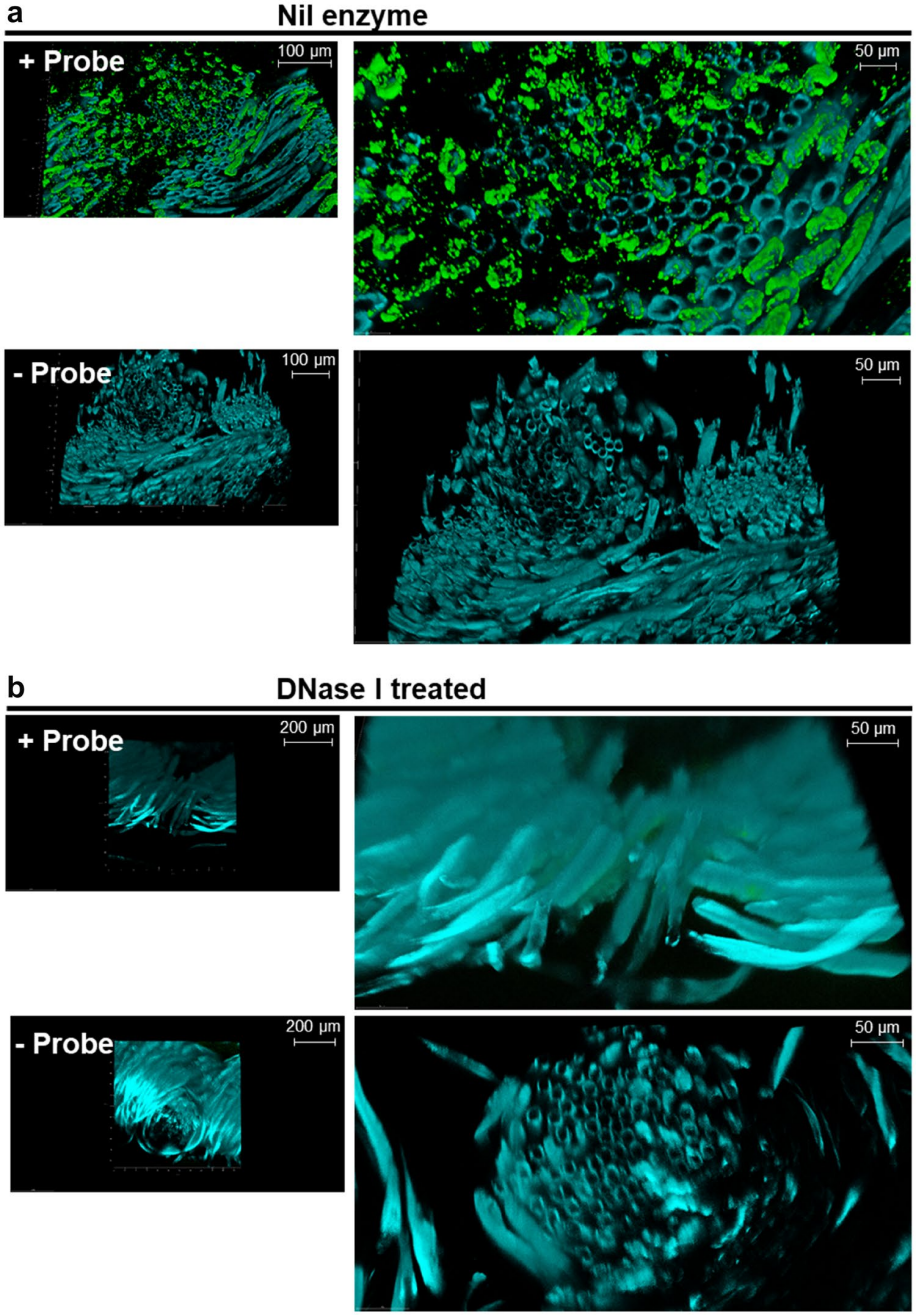
Addition of DNase I can remove eDNA embedded in-between textile fibers. Cross-sections of soiled cotton T-shirts washed with and without DNase I were probed with α-Z-DNA-ATTO^488^, to visualize eDNA embedded between the fibers. Color rendering—Cyan, fabric background; green, eDNA. (**a**) Cross sections of consumer T-shirts washed in Nil enzyme detergent, probed with and without α-Z-DNA-ATTO^488^. (**b**) Cross sections of soiled T-shirts washed in DNase I containing detergent probed with and without α-Z-DNA-ATTO^488^.

Washed swatches of T-shirt samples were set in an epoxy resin and hardener before being sliced using a vibratome to produce 150 µm transverse sections of the respective T-shirts. The cross-sections were probed with α-Z-DNA-ATTO^488^ and confocal microscopy was carried out to assess the extent of microbial eDNA deposition between the cotton fiber cells. In Nil enzyme washed T-shirt sections, there was a significant detection of ATTO^488^ signal which indicated the distribution of microbial eDNA to be predominantly around the cotton cell wall, as opposed to having fully penetrated through into the lumen of the cotton cells (Fig. 4a.). In contrast, there was minimal fluorescence signal on probed T-shirts, washed with DNase I (Fig. 4b). This suggests that the DNase I technology was not only conferring a DNA removal benefit on the surface of fabrics (S. Figure 4, Video attachment 1); but was also capable of cleaning deeper into the cotton yarn of consumer items, as shown by the discernibly lower levels of microbial eDNA remaining on T-shirt cross-sections washed with DNase I (Fig. 4).

## Discussion

Laundry relevant bacteria can be introduced onto textiles through wearing, washing and laundering processes^1,26–28^. The bacteria eventually build up biofilms and studies showed that an average 0.22 ± 0.1 g of eDNA per gram of cellular DNA, is produced by *Pseudomonas* biofilms^29^. This eDNA not only serves as a source of nutrients for biofilm sustenance and growth^30^, but has recently been shown to have roles in the establishment and structural integrity of other biofilm components which contribute to recalcitrance of soils on textiles^6,31^. It is now becoming clear that eDNA is a critical component of the EPS matrix and that EPS on laundry are not efficiently being removed in current wash conditions, using non biological detergents to counteract these soils of microbial origin^18^.

Bacteria are known to produce and actively secrete eDNA, or undergo mediated autolysis resulting in the lysed cells releasing eDNA^32–34^. Secreted outer membrane vesicles from bacteria are also known to commonly contain DNA, alongside other lipopolysaccharide and protein components^35^. These processes can all contribute to an increase in eDNA levels and hence the adhesion of other EPS components^36^. Besides the canonical form of B-DNA, DNA can also exist in other helical confirmations such as Z-DNA^37,38^. Z-DNA is a left-handed helical form of double-stranded DNA which forms behind active RNA polymerases, during transcription, to relieve torsional stress^37^. It is feasible that intracellular Z-DNA, amongst the other confirmations of DNA, are released as part of either quorum sensing-dependent or independent mechanisms^39,40^. Alternatively, it’s possible that genomic B-DNA released through these processes, could interconvert to Z-DNA when released onto the textile environment^41–43^.

Though usually only constituting a limited level of genomic DNA content^38,44^, we have found that α-Z-DNA is allowing us to detect and visualize a representative amount of microbial DNA on textiles, which corresponds to the quantitative reduction in eDNA on real items washed with DNase I. It could be that due to the increased exposure of laundry-residing bacteria to UV radiation; Z-conformation DNA is favored due to its resistance to intercalating agents with potential to cause further DNA damage^45^. Or perhaps in response to hot conditions or during exercise, the wearer’s body alters its osmoregulatory processes; which results in an increased salt (Na^+^) concentration on the worn garment due to an increased sweating response^46^. Z-DNA is reported to more readily form in the presence of salts or metal complexes and the deposition of Z-DNA alongside other EPS from laundry dwelling microbes could be reflecting that^24,41,47^. It’s also possible that due to the recognition specificity of the Z-DNA antibody, being the phosphodiester backbone of CG purine-pyrimidine base sequences, there is some general recognition of dsDNA by this antibody^24^ (Z-DNA formation is proportionally associated with high GC base content^48^). Further studies will be needed to elucidate the exact reason, but our current work suggests that the presence of Z-DNA in the EPS matrix might be prevalent, hinting at a possible role for this form of DNA in biofilm formation.

Conventional dogma has been that washing at high temperatures for longer times, with non-biological detergents would be enough to sufficiently clean our laundry. In the past years however, consumer attitudes and understanding have changed and there is a shift in laundering approaches to using lower temperatures and shorter wash times to reduce energy consumption. Consistent with these “green” initiatives; there is a need to identify new effective enzyme technologies which are sustainable and come with lower carbon manufacturing footprints.

The performance of current detergents at sustainable conditions (i.e. 40 °C for 30 min; typical European wash conditions), leave a lot of scope for improvement when it comes to tackling the sources of these undesirable characteristics^49^. Previous work has found that there are qualitative appearance benefits when adding DNase I for real consumer item cleaning; which translates into strong visual preferences for DNase I washed items^18^.

Our results support the hypothesis that addition of DNase I enzyme through the wash is an effective approach for removing microbial eDNA in textiles^18^. It is likely that some of the previously reported malodor and visual benefits brought about by DNase I addition are due to the quantitative reduction of eDNA^18^. However, to obtain deeper insight into the processes involved in eDNA accumulation on (and removal from) fabrics, it is necessary to be able to directly visualize these processes on real laundry samples. Whereas previous work has relied on detection of extracted DNA^50^, our approach using highly specific molecular probes enables in situ analysis of microbially derived eDNA species, Z-DNA.

In the present work, we visualized the removal of eDNA from garments through the addition of DNase I to a laundry detergent formulation. The effectiveness of DNases in disrupting soils of microbial origin are understood to be dependent on maturity of the microbial community^51^. It’s hypothesized that as such communities mature, eDNA becomes ‘protected’ by other exo-polymeric components, which results in de-sensitization of the macromolecular matrix to this treatment^31^. We hypothesize that in a washing machine context, together with the high levels of physical agitation exerted on our wash loads, there is ample disruption of these other components to enable DNase I to access previously sheltered eDNA residing deep in the fabric; and confer the eDNA removal and washing benefits which we see in our current study and in previous studies^18^.

There are also potential therapeutic applications of DNase technology beyond fabric-care. The addition of DNase I to remove eDNA has been shown to increase the susceptibility of biofilms to cationic aminoglycosides^52–54^ and in reducing the viscosity of cystic fibrosis sputum^55^. Its use has also been investigated for household surface and food industry cleaning applications^56^. This further emphasizes the value of increasing our understanding of DNases and refining their formulation for uses in various contexts. The use of additional molecular probes (i.e. monoclonal antibodies, aptamers or lectins) could also be leveraged in a similar approach to visualize removal of other soils of microbial origin from textiles. Hence further work will be done to explore and expand our library of molecular probes to help support such applications.

In conclusion, the current work further demonstrates the benefit of the inclusion of a new-to-laundry DNase in detergent formulations^18^. We present DNA extraction data alongside epifluorescence and confocal laser scanning microscopy images which support a quantitative and visible benefit of DNase I addition, to microbially-derived eDNA removal in real items.

## Materials and methods

A list of key materials, equipment and suppliers are provided in S. Table 2.

### Washing conditions

Fabric items were washed in front-loading Miele washing machines (W1714) for 1 h 25 min at 40 °C, using city water representative of UK water hardness of 18–20 grains per US gallon (gpg) with Ariel 3in1 Pods Original; 1 pod per wash (> 30% anionic surfactants, 5–15% soap; < 5% nonionic surfactants, phosphonates, enzymes, optical brighteners, perfumes, alpha‐isomethyl ionone, citronellol, coumarin, and linalool). Nuclease enzyme (DNase I, E.C.3.1.21.1) was used at 0.5 ppm active enzyme protein in the wash alongside 2 kg of clean cotton and polyester ballast and 4 × SBL2004 soil sheets. Washed fabrics were dried in a tumble dryer before further analysis.

### DNA extraction and PicoGreen^®^ quantification assay

DNA was extracted from 3.5 g of respective garment swatches with DNA extraction solution (10 mM EDTA, 0.9% (w/v) NaCl); using a shaker plate set to 40 °C, for 2.5 h at 900 RPM agitation. Spectrophotometric DNA quantification was done using commercially available PicoGreen (1:20,000 dilution) (Thermo Fischer Scientific, Life Technologies, P7581) with a serial dilution series of Herring sperm DNA (Sigma, CAS no.: 68938-01-2); starting at 1 mg/ml–1 ng/ml used as standard^57,58^.

### Immunocytochemistry procedures

Sample probing and imaging was carried out as previously described^59^, with changes. Multiple 5 × 1 cm to 1 × 1 cm sections were cut from the central back region of corresponding T-shirts and the mid-section of towels and placed into 12–24 well plates.

Anti-Z-DNA (Z22) recombinant monoclonal antibody with custom ATTO^488^ conjugate (Absolute Antibody) was diluted 100–200-fold in phosphate-buffered saline (PBS) containing 5% (w/v) milk protein (MP-PBS). Controls were incubated in MP-PBS only. Fabric samples were incubated with respective solutions at ambient room temperature on a Cole-Parmer Stuart™ orbital rocker plate, set to agitate at 50 RPM, for 1.5 h. Samples were then washed in PBS containing 0.1% (w/v) Tween-20 (PBS-T) once, for 5 min, in darkness; before a further two times in 1 × PBS only, again for 5 min in darkness. Citifluor AF1 anti-fade mounting solution (Electron Microscopy Sciences) was added to each sample once placed on microscope slides and secured with 1.7 × 2.8 cm adhesive Gene frames (ThermoFisher scientific).

Epi-fluorescence imaging was carried out on a Leica DM6B upright digital research microscope with CTR6 LED electronics box. Image acquisition was carried out using Leica Application Suite X software (3.4.2.18368.1.2) using a fluorescence filter channel covering wavelengths of 480 nm (excitation) and 520 nm (emission). Samples were exposed for 50–200 ms and images captured using 1.25×, 5× and 10× objectives.

Specificity of probe binding to DNA was ascertained by treating samples with DNA decontamination reagent (Sigma-Aldrich, 43944) for 1 h at ambient room temperature prior to incubation with α-Z-DNA-ATTO^488^ and imaging as described above.

Confocal laser scanning imaging was carried out on a Leica SP8 confocal microscope equipped with a 20 × 0.75 NA objective, using 405 nm and 488 nm lasers for fiber autofluorescence and ATTO^488^ fluorescence, respectively. Z stack images were captured of random areas of the samples of between 60 and 120 µm and rendered using LasX software.

### Methyl-Green post wash reveal assay

Methyl Green (Sigma, M8884-5G) solution was prepared at 1 mg/ml in deionized water. Respective fabric swatches (clean, unwashed, Nil enzyme, DNase I washed) were incubated in methyl-green solution for 5 min before being washed in clean deionized water and left to dry at ambient room temperature. Images of swatches were captured using a high-resolution scanner (Canon, 9000F Mark II).

### Immunoblot assay

Protocol for immuno(dot)blot assays on nitrocellulose filter was adapted^60^ and modified for fabric textiles. Briefly, 0.5 cm × 0.5 cm grids were drawn onto 0.45 µm nitrocellulose membrane (Amersham™) or clean cotton fabric (Warwick Equest Limited), with pencil or permanent marker respectively. Ten-fold serial dilutions of microbial DNA (D8259, highly polymerized, high GC content, Sigma) and Herring sperm DNA (D6898, Sigma) prepared at concentrations of 1 mg/ml–1 ng/ml were spotted onto nitrocellulose and fabric respectively along with a control serial dilution of PBS (VWR, K813-500 ml) and left to dry at ambient room temperature for 24 h. Nitrocellulose membrane and fabric blot samples were incubated with α-Z-DNA antibody (Absolute Antibody) diluted 100 to 200-fold in phosphate-buffered saline (PBS) containing 5% (w/v) milk protein (MP-PBS). Blots were washed in 1 × PBS containing 0.1% (w/v) Tween-20 (PBS-T) once, for 5 min; before a further two times in 1 × PBS only, again for 5 min with agitation.

Samples were then incubated with alkaline phosphatase conjugated goat α-mouse secondary antibody (Jackson ImmunoResearch, 115-055-068), diluted 1 in 5000 in 1 × PBS supplemented with 5% (w/v) skimmed milk powder, for 1.5 h at room temperature on an orbital shaker. Blots were washed a final time as described above.

Binding of antibodies to nitrocellulose and fabric immunoblots was evaluated using alkaline phosphatase staining. Blots were developed in a solution containing 5-bromo-4-chloro-3-indolylphosphate (Melford, B74100-1.0) and nitro blue tetrazolium (Melford, N66000-1.0) in alkaline phosphatase buffer (100 mM NaCl, 5 mM MgCl_2_, pH 9.5)^61^.

### Sample sectioning (epoxy resin fixing)

Sample swatches measuring 5 cm × 1 cm were cut from the central back region of corresponding T-shirts and embedded in a 50:50 mix of Diall Epoxy resin and Diall hardener for epoxy resin (batch L.1921703). After 30 min hardening, the resin block was mounted on a metal plate for vibratome sectioning (7000smz-2 vibrating microtome, Campden Instruments). Repeated sections of 150 µm were automatically cut with a stainless-steel blade in a bath of distilled water.

## Supplementary Information

Supplementary Figure 1.

Supplementary Figure 2.

Supplementary Figure 3.

Supplementary Figure 4.

Supplementary Legends.

Supplementary Table 1.

Supplementary Table 2.

Supplementary Table 3.

Supplementary Video 1.
