# Supplementary material for: Removal of eDNA from fabrics using a novel laundry DNase revealed using high-resolution imaging
Source: Sci Rep. 2021 Nov 2;11:21542. doi: 10.1038/s41598-021-98939-0 (PMC8563969; doi:10.1038/s41598-021-98939-0)
Supplement: Supplementary file 1 — Supplementary Figure 1. [file 41598_2021_98939_MOESM1_ESM.docx]

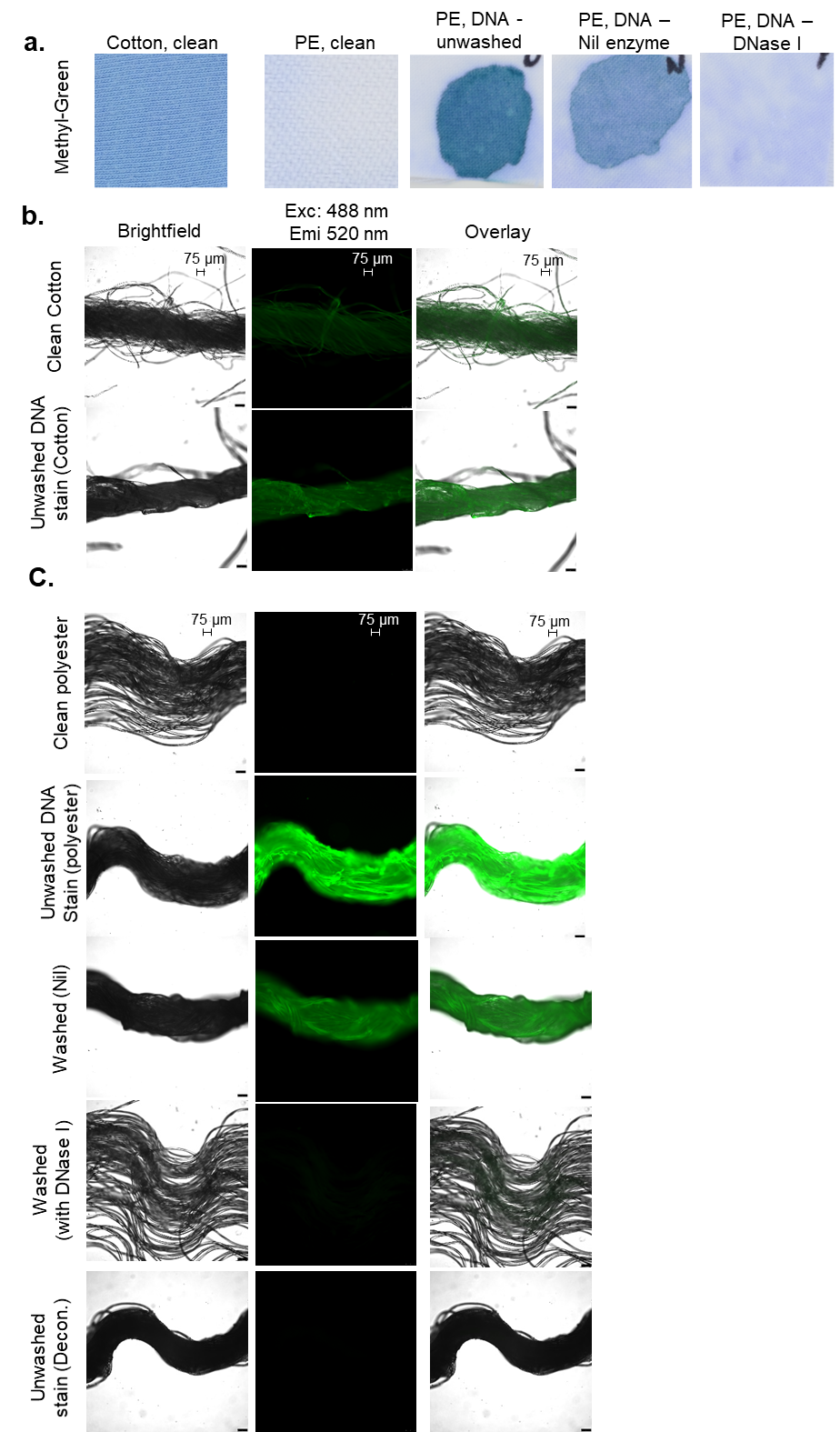


**Supplementary figure 1 – Incompatibility of DNA stains with cotton poses a problem for visualizing DNA removal by DNase I on cotton fabrics but not on polyester fabrics**

**a**. Methyl Green staining of cotton gave rise to a high level of background; but can be used to visualise DNase I effectiveness against herring sperm DNA. spiked onto polyester.

**b**. PicoGreen staining of cotton threads from swatches spiked with 1 mg/ml herring sperm DNA show similar levels of fluorescence when imaging using excitation/emission wavelengths 480/520, respectively.

**c**. PicoGreen staining of Polyester threads from swatches spiked with 1 mg/ml herring sperm DNA; show reduction of eDNA after washing with DNase I. Fluorescence microscopy carried out using excitation/emission wavelengths 480/520, respectively. Decon, DNA decontaminant solution.
