# Supplementary material for: Removal of eDNA from fabrics using a novel laundry DNase revealed using high-resolution imaging
Source: Sci Rep. 2021 Nov 2;11:21542. doi: 10.1038/s41598-021-98939-0 (PMC8563969; doi:10.1038/s41598-021-98939-0)
Supplement: Supplementary file 2 — Supplementary Figure 2. [file 41598_2021_98939_MOESM2_ESM.docx]

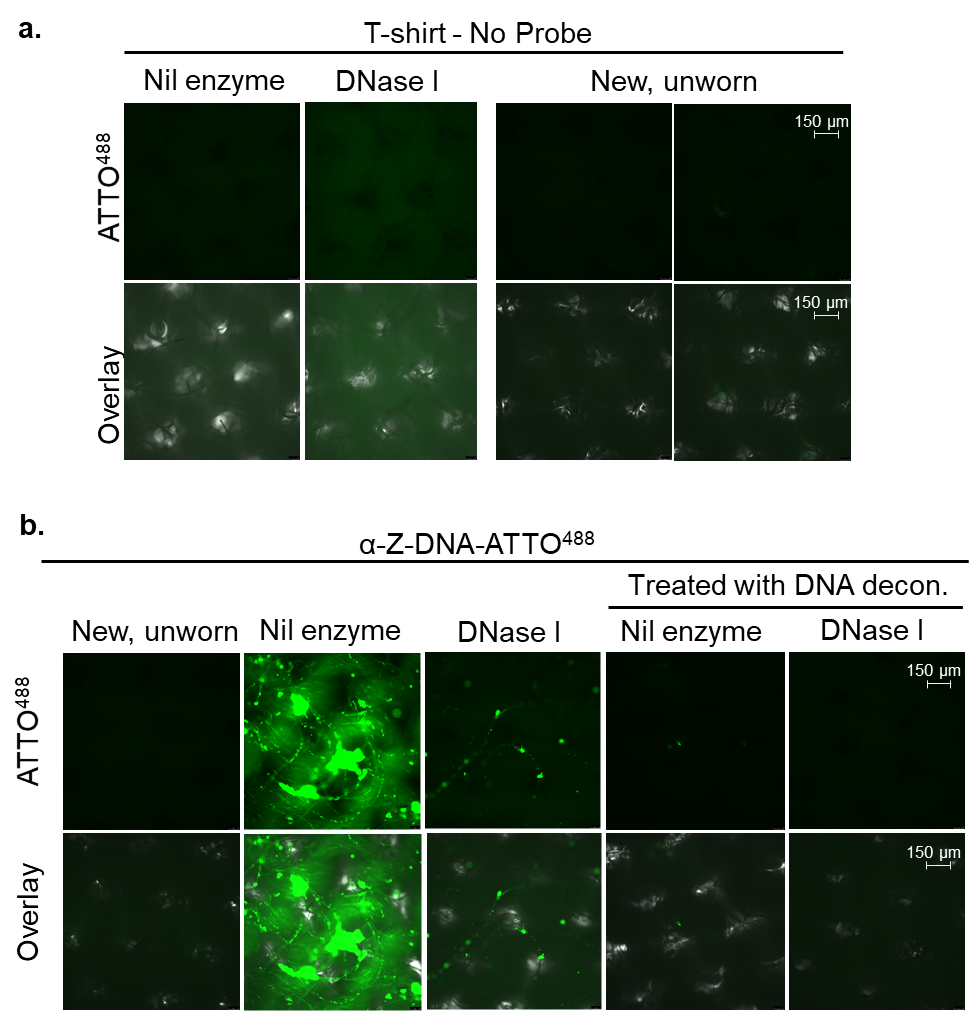


**Supplementary figure 2 – Fluorescence observed on probed T-shirts is not due to autofluorescence of fabric and is specific to binding of molecular probe to eDNA deposited on the garment during use**

**a**. Fluorescence microscopy imaging of a soiled and equivalent new unworn T-shirt in the absence of α-Z-DNA-ATTO^488^, taken at 10x magnification.

**b**. T-shirt samples pre-incubated with and without DNA decontaminant solution and subsequently probed with α-Z-DNA-ATTO^488^, imaged at 10x magnification.
