# Supplementary material for: Removal of eDNA from fabrics using a novel laundry DNase revealed using high-resolution imaging
Source: Sci Rep. 2021 Nov 2;11:21542. doi: 10.1038/s41598-021-98939-0 (PMC8563969; doi:10.1038/s41598-021-98939-0)
Supplement: Supplementary file 3 — Supplementary Figure 3. [file 41598_2021_98939_MOESM3_ESM.docx]

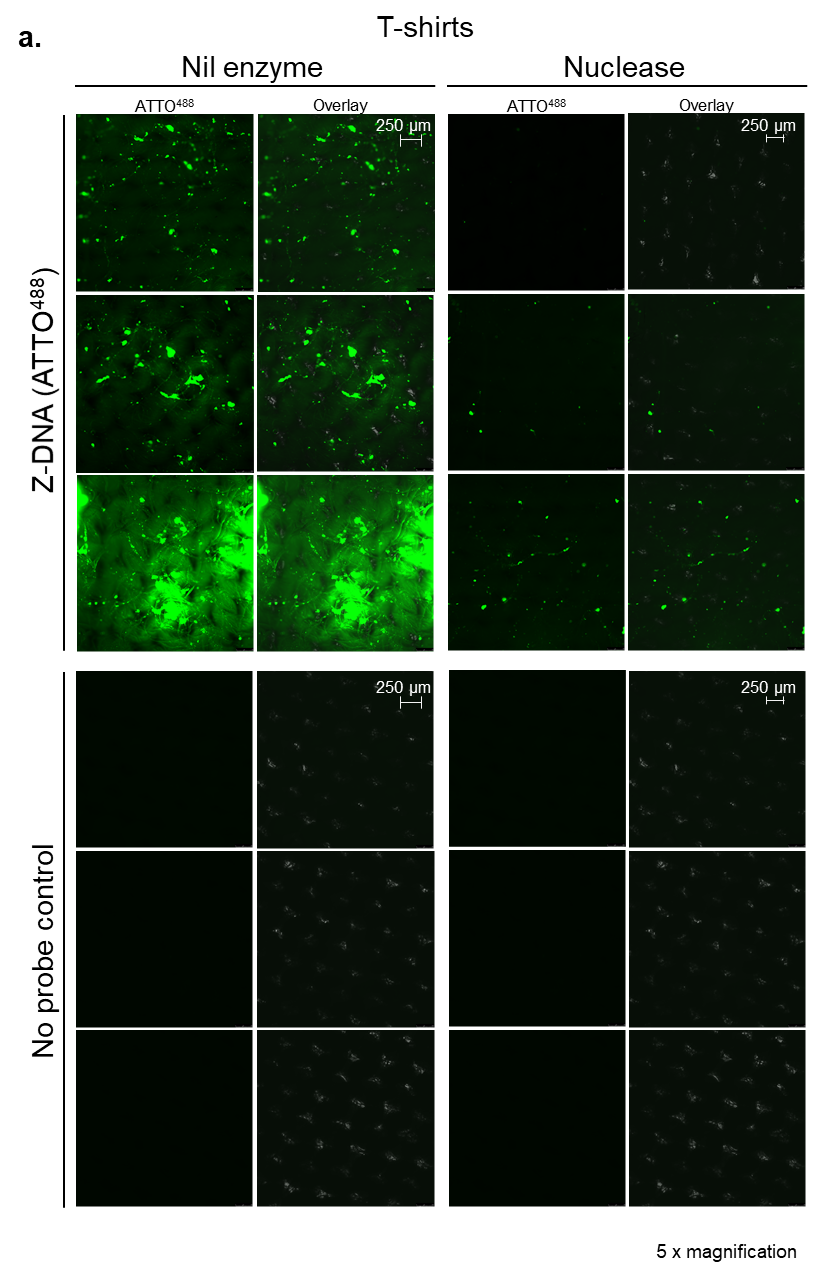


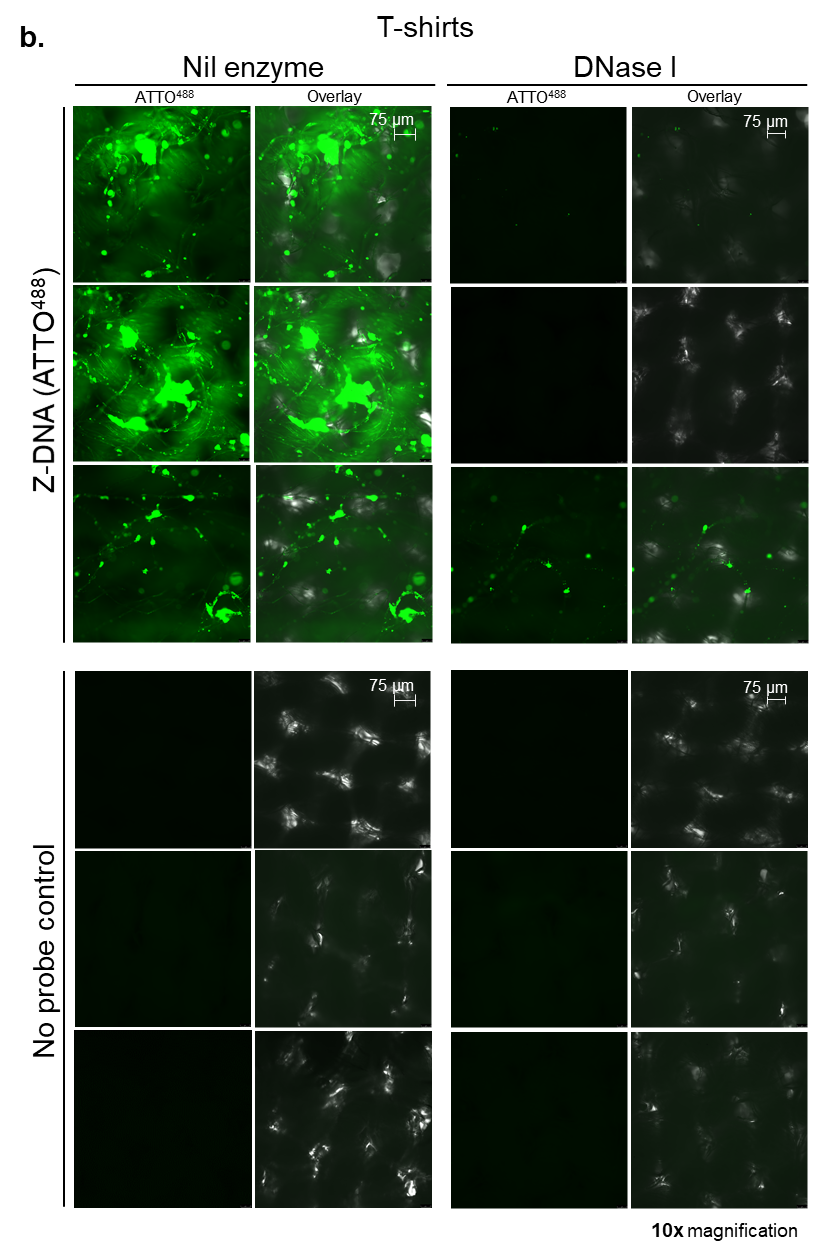


**Supplementary figure 3** – **T-shirts washed in the presence of a new-to-laundry DNase consistently show a greater reduction in fluorescence, corresponding to more efficient eDNA removal**

Further fluorescence microscopy images of soiled T-shirts washed in Nil enzyme *vs.* DNase I containing detergent, probed with and without α-Z-DNA-ATTO^488^ . Imaged at **a.** 5x and **b.** 10x magnifications, respectively. ATTO^488^ spectrum Exc: 480 nm; Emi: 520 nm; 200 ms exposure time. Fluorescence density quantified in S. Table 3.
