# Supplementary material for: Removal of eDNA from fabrics using a novel laundry DNase revealed using high-resolution imaging
Source: Sci Rep. 2021 Nov 2;11:21542. doi: 10.1038/s41598-021-98939-0 (PMC8563969; doi:10.1038/s41598-021-98939-0)
Supplement: Supplementary file 4 — Supplementary Figure 4. [file 41598_2021_98939_MOESM4_ESM.docx]

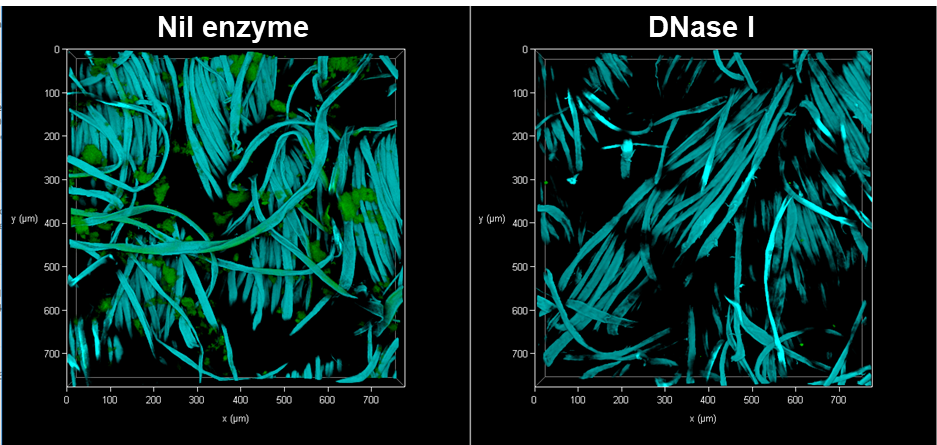


**Supplementary figure 4.** - Confocal microscopy was used to visualize binding of α-Z-DNA-ATTO^488^ probe to eDNA remaining on consumer worn cotton T-shirts washed in Nil enzyme (left) or in presence of DNase I (right). Cyan, fabric background; green, eDNA. Playable video link in video attachment 1
