# Supplementary material for: Removal of eDNA from fabrics using a novel laundry DNase revealed using high-resolution imaging
Source: Sci Rep. 2021 Nov 2;11:21542. doi: 10.1038/s41598-021-98939-0 (PMC8563969; doi:10.1038/s41598-021-98939-0)
Supplement: Supplementary file 5 — Supplementary Legends. [file 41598_2021_98939_MOESM5_ESM.docx]

S. Table 1 – PicoGreen DNA quantification on T-shirts and pillowcases

S. Table 2 – List of Key equipment, materials and suppliers

S. Table 3 – Relative Fluorescence quantification of probed Towels and T-shirts Nil enzyme vs. DNase I

Video attachment 1 – Confocal microscopy video (Nil vs DNase I)
